# Re-evaluating a vision-related quality of life questionnaire with item response theory (IRT) and differential item functioning (DIF) analyses

**DOI:** 10.1186/1471-2288-11-125

**Published:** 2011-09-02

**Authors:** Ruth MA van Nispen, Dirk L Knol, Maaike Langelaan, Ger HMB van Rens

**Affiliations:** 1 Department of Ophthalmology, VU University Medical Center, Amsterdam, the Netherlands; 2 EMGO+ Institute for Health and Care Research, VU University Medical Center, Amsterdam, the Netherlands; 3 Department of Epidemiology and Biostatistics, VU University Medical Center, Amsterdam, the Netherlands; 4 Netherlands Institute for Health Services Research (NIVEL), Utrecht, the Netherlands; 5 Department of Ophthalmology, Elkerliek Hospital, Helmond, the Netherlands

**Keywords:** Visual impairment, Vision-related quality of life, Item response theory, Graded response model, Differential item functioning

## Abstract

**Background:**

For the Low Vision Quality Of Life questionnaire (LVQOL) it is unknown whether the psychometric properties are satisfactory when an item response theory (IRT) perspective is considered. This study evaluates some essential psychometric properties of the LVQOL questionnaire in an IRT model, and investigates differential item functioning (DIF).

**Methods:**

Cross-sectional data were used from an observational study among visually-impaired patients (n = 296). Calibration was performed for every dimension of the LVQOL in the graded response model. Item goodness-of-fit was assessed with the S-X^2^-test. DIF was assessed on relevant background variables (i.e. age, gender, visual acuity, eye condition, rehabilitation type and administration type) with likelihood-ratio tests for DIF. The magnitude of DIF was interpreted by assessing the largest difference in expected scores between subgroups. Measurement precision was assessed by presenting test information curves; reliability with the index of subject separation.

**Results:**

All items of the LVQOL dimensions fitted the model. There was significant DIF on several items. For two items the maximum difference between expected scores exceeded one point, and DIF was found on multiple relevant background variables. Item 1 'Vision in general' from the "Adjustment" dimension and item 24 'Using tools' from the "Reading and fine work" dimension were removed. Test information was highest for the "Reading and fine work" dimension. Indices for subject separation ranged from 0.83 to 0.94.

**Conclusions:**

The items of the LVQOL showed satisfactory item fit to the graded response model; however, two items were removed because of DIF. The adapted LVQOL with 21 items is DIF-free and therefore seems highly appropriate for use in heterogeneous populations of visually impaired patients.

## Background

The detrimental effects of living with vision loss caused by irreversible eye conditions (such as age-related macular degeneration or diabetic retinopathy) are well reported [1]. Research in low vision has primarily focused on older adult populations, because of increased prevalence of age-related eye conditions in older age [2-8]. Those studies used several vision-related quality-of-life questionnaires which allow to assess the disability suffered in daily life [9,10]. In their review, de Boer et al. reported that the original Low Vision Quality Of Life questionnaire (LVQOL) was one of the best for use in patients with low vision [11,12]; its items are mainly related to difficulties people have in performing certain activities due to their visual disability. In a few studies within the framework of classical test theory, de Boer et al. translated and further validated the Dutch version of the LVQOL [13,14]. In two subsequent studies on the longitudinal outcomes of low vision rehabilitation, additional comments on the validity of the LVQOL were made using item response theory (IRT); however, a calibration-process was not performed [5,15]. In these studies, which were performed on the data previously used by de Boer et al. [13,14], it was concluded that on the dimension "Reading and fine work", the item invariance assumption did not hold over time. The lack of item invariance might have been a redundant phenomenon if the items had been calibrated in an IRT model beforehand.

Nowadays, IRT models are recommended for evaluating patient-reported outcomes; some questionnaires have been re-evaluated using the Rasch model [9,16-19], which is considered a special case of an IRT model [20]. IRT models represent a collection of statistical models for item analysis in questionnaires that measure a latent construct, i.e. vision-related quality of life, and for estimating individual scores for the construct, based on responses to the items. Another IRT model is the graded response model (GRM), which is a cumulative probability model. Although the Rasch model has favorable measurement properties, such as statistical sufficiency and specific objectivity, it is often too restrictive, especially for existing tests (developed in the classical test theory framework). For evaluative purposes, less constrained models such as the GRM often give a more realistic reflection of the data compared to Rasch or partial credit models [20]. Furthermore, from studies on cognitive processing in which it is investigated how response options are chosen, the GRM seems most appropriate for Likert-type items [21-23]. Another advantage of the GRM is that although a normal distribution of the latent variable is assumed, the model is quite robust to slight deviations from normality [24,25].

In an IRT calibration process some steps need to be taken, such as assessing item fit and differential item functioning (DIF) [26]. A large proportion of items with DIF is a severe threat to its construct validity and thus to the ability to draw conclusions based on the test scores [27]. Variables that potentially lead to DIF are demographic variables. A DIF analysis allows to examine the relationship between item responses and another variable, such as gender or age group, conditional on a measure of the latent construct, i.e. vision-related quality-of-life [28]. Disease-related variables may also lead to DIF, e.g. items may be interpreted differently by patients with different eye conditions, but with a similar disability level. Although vision-related quality-of-life questionnaires measure at the disability level, items could be problematic to patients in different ways due to differences in visual impairment, such as visual acuity or field loss. This could indicate whether there should be separate calibrations for populations with specific eye conditions [10] or demographic variables.

Since the LVQOL has not yet been calibrated, it remains unknown whether the items appropriately fit an IRT model. Therefore, the present study evaluates some essential psychometric properties of the LVQOL, including assessing item goodness-of-fit and DIF between subgroups.

## Methods

### Design and participants

Cross-sectional data were obtained from a longitudinal study: i.e. visually impaired older patients of an observational study on the vision-related quality-of-life effects of two types of low-vision rehabilitation (optometric service and multidisciplinary rehabilitation service) [4,10]. Consecutive patients (n = 357) were recruited from the ophthalmology departments of four hospitals in the Netherlands between July 2000 and January 2003. The eligibility requirements for inclusion in the study were referral to either the optometrist or the multidisciplinary low-vision service by an ophthalmologist, age over 50 years, no previous contact with low-vision rehabilitation services, irreversible vision loss, adequate understanding of the Dutch language, and adequate cognitive abilities. Patients who met the inclusion criteria were informed about the study and were invited to participate. From the eligible patients 17.1% did not participate. Baseline data were available of 296 visually impaired patients. Written consent was obtained from all participants. The study protocol was approved by the Medical Ethics Committee of the VU University Medical Center, and conducted according to the principles of the Declaration of Helsinki.

### Measurements

#### Patient characteristics

Demographic variables and other characteristics (e.g. age, gender and main cause of vision loss) were taken from the patients' hospital charts. Rehabilitation type was either the optometric, or multidisciplinary service. Distance visual acuity was assessed for all participants by their ophthalmologist by projection and with habitual correction for both eyes separately. To enable meaningful computations, decimal visual acuity values were transformed to logMAR values, where higher values represent more vision loss, or lower visual acuity values.

#### Vision-related quality-of-life

The LVQOL was previously forward and backward translated by two different native speakers on separate occasions. Few dissimilarities were resolved [13]. In the present study, the Dutch version of the LVQOL was re-evaluated. The questionnaire was in large print and was completed by the patients either independently or with assistance from others. The 25 items on the LVQOL are mainly related to difficulties people have in performing certain activities due to their visual disability, ranked on a 6-point Likert-type scale: 0 "No problem" to 5 "Not able because of vision". In our previous study two items were removed from the questionnaire [5], therefore this report is based on 23 items.

### Validation and statistical analyses

#### Assessing dimensionality and local independence

Unidimensionality is a critical assumption of IRT. It refers to whether a person's response to an item that measures a construct is accounted for by the level on that trait, and not by other factors [29]. In a previous study, dimensionality of the LVQOL was investigated on baseline data of the low-vision rehabilitation effect study [5]. In summary, an exploratory factor analysis on polychoric correlations and Promax rotation in Mplus version 3.13 was carried out. The model parameters were estimated applying weighted least squares with mean and variance correction (WLSMV). Item 5 "Problems reading street name signs" and item 25 "Problems doing household tasks" had low factor loadings and interpretation of factors was confusing (both items loaded almost equally on two factors). After removing items 5 and 25, the factor analysis yielded four dimensions: "Mobility", "Reading and fine work", "Adjustment" and "Basic aspects" (explained variance 75%). The root mean-square residual, which is an index of global model fit, was satisfactory: i.e. 0.03 and, factor loadings were all higher than 0.40. The Cronbach's alpha-values for these were 0.84, 0.90, 0.82 and 0.93, respectively.

To further prepare for the IRT analyses, we assessed local independence of items by inspection of possible excess correlation among items in the residual correlation matrix. Local dependence could arise from items with a similar content or wording. Inspection of the residual correlations showed that it was highest between items 17 "Reading large print" and 24 "Using tools" (-0.11), but the other residual correlations were never higher than 0.09 and were therefore not considered to be a problem. The psychometric properties of the LVQOL dimensions were further assessed with an IRT model.

#### IRT calibration

In the present study, we used the GRM to evaluate the LVQOL [30], which is a generalization of the two-parameter logistic model.

In the GRM, the cumulative probability (P*) of responding in category *j *or higher on item *i *of a person *s *with disability *θ_s_*, i.e. the 'underlying' or 'latent' variable, is given by

$$P_{ij}^{*}\left(\theta_{s}\right)=\frac{\exp\left[\alpha_{i}\left(\theta_{s}-\beta_{ij}\right)\right]}{1+\exp\left[\alpha_{i}\left(\theta_{s}-\beta_{ij}\right)\right]},$$

with item parameters *α_i _*as the slope or discrimination parameter and *β_ij _*as the threshold or difficulty parameters of item *i*. A high *α_i _*indicates that the response categories differentiate well across disability levels [20]. Each item (*i*) on (a dimension of) the LVQOL is described by one α*_i_*, and by five *β_ij_*, which is one less than the number of response categories. The point along the disability continuum at which respondents have a 0.50 probability of endorsing response category *j *or higher of item *i *is represented by *β_ij_*. From the P*, the probability of endorsing category *j *of item *i *is obtained by

$$P_{ij}^{}\left(\theta_{s}\right)=P_{ij}^{*}\left(\theta_{s}\right)-P_{i,j+1}^{*}\left(\theta_{s}\right).$$

It is assumed that the prior distribution of the person parameter (*θ_s_*) is standard normal (mean 0; SD 1) [20]. The item parameters were estimated in MULTILOG by the method of marginal maximum likelihood [31]. Subsequently, posterior estimates of *θ_s _*can be obtained.

Even after unidimensionality and local independence have been investigated, some items might have remained that do not fit the GRM. Applications of IRT implicitly assume that the model is correct; that is, expected item scores should increase monotonically and the item response model should reflect the data accurately. Although a certain amount of misfit is inherent to every model, considerable misfit should be avoided. Item fit can be examined by comparing model predictions (expectations) and observed data [20]. By using item tests, decisions can be made as to whether it is necessary to remove any items. Therefore, item goodness-of-fit was investigated with an item test by Bjorner et al. [32], which is implemented in SAS [31,32]. This item-test is an extension (generalization) of the item test with dichotomous response categories which was developed by Orlando and Thissen and is known as the S-X^2^-test [33,34]. Items were considered as misfitting to the model if p < 0.01.

Examining DIF is important in the investigation of the equivalence of items across subgroups differing in background characteristics [28,35]. We investigated DIF on the subgroup variables age (arbitrarily chosen > or ≤75 years), gender (male versus female), main cause of vision loss in the best eye (age-related macular degeneration versus other eye conditions), rehabilitation type (optometrist versus multidisciplinary service), logMAR visual acuity level (≥ 0.52; low vision/blindness or < 0.52; mild vision loss), and types of administration (self-reported versus assisted by a significant other who filled out the questionnaire together with the patient). Two types of DIF were investigated: uniform DIF indicates that the item bias is in the same direction at all levels of the disability continuum, where one subgroup seems to have a consistently higher or lower likelihood to respond favorably to an item compared to its counterpart. In contrast to items with dichotomous response categories, for polytomous items this may vary for every *β_ij_*, i.e. without affecting *α_i_*. Non-uniform DIF indicates dissimilarity in *α_i _*between subgroups, conditional on the disability level, which reflects subgroup by ability interaction [28]. DIF analyses were performed with software for the computation of statistics involved in IRT likelihood-ratio tests for DIF (IRTLRDIF) by Thissen [36,37]. This approach tests the null hypothesis that *α_i _*is equal for two subgroups (absence of non-uniform DIF), yielding a Chi-square (G^2^) statistic with one degree of freedom, and the null hypothesis that the *β_ij _*is equal (absence of uniform DIF) between subgroups, using five degrees of freedom. IRTLRDIF is based on a hierarchical structure, which means that *β_ij _*is tested for uniform DIF, only if the test for *α_i _*is not significant. To correct for multiple testing, a p-value < 0.01 was indicated as statistically significant occurrence of DIF.

To gain more insight into DIF items (particularly to examine the magnitude of DIF between subgroups), we calculated differences in expected scores for those subgroups. The magnitude of DIF was presented as the maximum difference between expected scores. When DIF cannot be resolved, a solution would be to separately estimate item parameters for subgroups; those parameters can subsequently be used to estimate the person parameter (*θ_s_*) [38]. Another solution is to remove the item. In the present study, items were removed on the basis of the magnitude of DIF which was determined by a large difference (> 1 point) between expected scores on the item; if there was DIF between more than one subgroup variable; or if DIF was present on a relatively large part of the disability continuum. After removing DIF items, the dimensions of the LVQOL were re-calibrated and DIF analyses were repeated to see whether other DIF items would resolve. Subsequently, 'test information' was presented for the dimensions of the LVQOL. Test information refers to the range of the underlying construct over which (a dimension of) a test is most useful to distinguish between respondents. Therefore, information represents the reliability or measurement precision. The inverse of the square root of the information function is equivalent to the standard error (SE) of *θ_s _*[24]. Test information for the separate dimensions of the LVQOL was analyzed in MULTILOG [31] and the corresponding curves presented. Finally, the reliability coefficient was calculated for *θ_s _*of the separate LVQOL dimensions (index of subject separation) [39].

## Results

### Patient characteristics

Table 1 presents the characteristics of the patients. Mean age was 78.4 (SD 8.8; range 52-98). Mean logMAR Visual Acuity was 0.67 (SD 0.39). Besides age-related macular degeneration (53%), the most common other causes of vision loss were diabetic retinopathy, glaucoma and cataract (47%). About 63% of the patients were assisted by a significant other with administering the LVQOL, i.e. 15.5% by their spouse, 40.9% by family members and 6.5% by a friend, a nurse or someone else.

**Table 1:** Patient characteristics (N = 296)

**Gender**		
Female	183	61.8%
Male	113	38.2%
		
**Age**		
< 75 years	76	25.7%
≥75 years	220	74.3%
		
LogMAR visual acuity*		
< 0.52 mild vision loss	97	32.9%
≥ 0.52 low vision/blindness	198	67.1%
		
Main cause of vision loss in best eye*		
Age-related macular degeneration	154	52.6%
Other eye conditions	139	47.4%
		
**Rehabilitation type**		
Optometric service	161	54.4%
Multidisciplinary service	135	45.6%
		
Administration type*		
Self-report	108	37.1%
Assisted	183	62.9%

* 1, 3 and 5 missing values

### Item non-response and goodness-of-fit

The item non-response was 4.1% for "Basic aspects" (60 missing responses for 5 items); 4.8% for "Mobility" (71 missing responses for 5 items); 4.1% for "Adjustment" (61 missing responses for 5 items); and 4.8% for "Reading and fine work" (113 missing responses for 8 items). The total item non-response for the LVQOL was 4.5%. All items of the four separate LVQOL dimensions fit the GRM.

### Differential item functioning

Table 2 presents items with DIF between different subgroups, meaning that there was interference between item responses of different subgroups at similar disability levels. For example, on the "Adjustment" dimension, item 1 'Vision in general' had uniform DIF on two subgroup variables, i.e. gender and administration mode. Patients who self-administered the questionnaire responded lower to this item (reflecting less disability) than patients who were assisted by a significant other, conditional on the disability level. Particularly at the higher extremity of the disability continuum (representing more disability), women responded lower to this item than men. This difference was caused by a small number of responses in the highest category. Furthermore, on the "Reading and fine work dimension", two items had DIF, i.e. item 19 'Reading labels' (uniform DIF between eye condition subgroups) and item 24 'Using tools, e.g. using a hammer or threading a needle' (uniform DIF between men and women) to which women responded higher than men, conditional on the disability level.

**Table 2:** Items with DIF between subgroups of relevant variables

	Item content	Subgroups	α	***β***_***1***_	*β_2_*	*β_3_*	*β_4_*	*β_5_*	**G**^**2**^	df	p	**ES**_**Δmax**_	*θ*
**Basic aspects**													
7.	Eyes getting tired	Low vision	2.50	-0.93	-0.65	-0.10	0.32	2.13	15.7	5	0.008	-0.80	-0.80
		Moderate vision loss	2.50	-1.37	-1.23	-0.54	0.10	1.86					
**Mobility**													
3.	Seeing steps or curbs*	Low vision	2.38	-0.97	-0.61	-0.01	0.21	1.56	14.2	1	< 0.001	-0.74	0.20
		Moderate vision loss	5.76	-0.96	-0.71	-0.26	-0.02	1.68					
**Adjustment**													
1.	Vision in general	Self	1.99	-1.98	-1.44	-0.14	0.38	2.33	19.4	5	0.002	-0.70	-2.00
		Assisted	1.99	-2.47	-2.30	-0.56	-0.09	2.33					
1.	Vision in general	Male	2.08	-2.05	-1.44	0.04	0.40	1.95	17.9	5	0.003	0.62	2.60
		Female	2.08	-1.83	-1.57	-0.27	0.24	3.37					
12.	Unhappy situation in life^†^	Male	3.27	-0.74	-0.31	0.56	1.03	3.06	15.4	5	0.009	1.00	5.40
		Female	3.27	-0.39	-0.27	0.51	0.88	7.57					
**Reading and fine work**													
19.	Reading labels	AMD	3.77	-1.34	-1.21	-0.94	-0.46	0.68	17.8	5	0.003	0.24	-0.80
		Other	3.77	-1.48	-1.16	-0.63	-0.51	0.68					
24.	Using tools	Male	1.83	-1.22	-0.92	-0.38	-0.15	1.13	35.2	5	< 0.001	-1.22	-1.00
		Female	1.83	-1.80	-1.58	-1.39	-0.94	1.05					

Person distribution standard normal; *α *= item discrimination parameter; *β *= item threshold parameter; G^2 ^= Chi-square statistic; df = degrees of freedom; ES_Δmax _= maximum difference in expected scores, *θ = *person parameter (disability level is presented at which the maximum difference in expected scores was found); AMD = age-related macular degeneration.

* Non-uniform DIF; † After deleting item 1, DIF for item 12 was not statistically significant.

**Figure 1:**
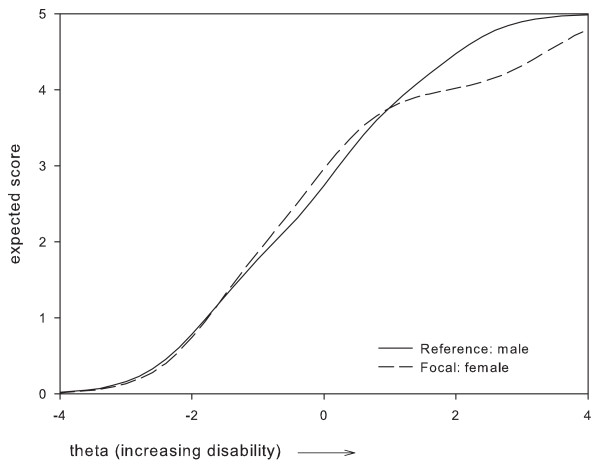
**Uniform DIF on item 1 'vision in general' between gender subgroups**.

**Figure 2:**
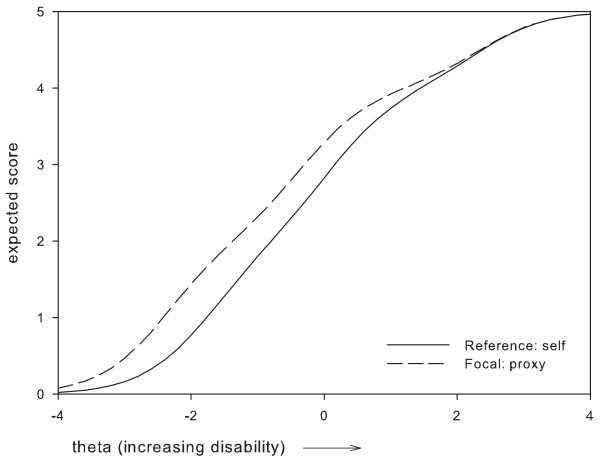
**Uniform DIF on item 1 'vision in general ' between administration mode subgroups**.

**Figure 3:**
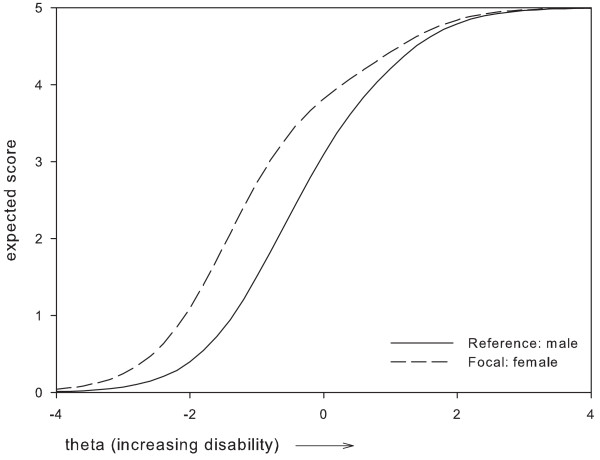
**Uniform DIF on item 24 'using tools' between gender subgroups**.

**Figure 4:**
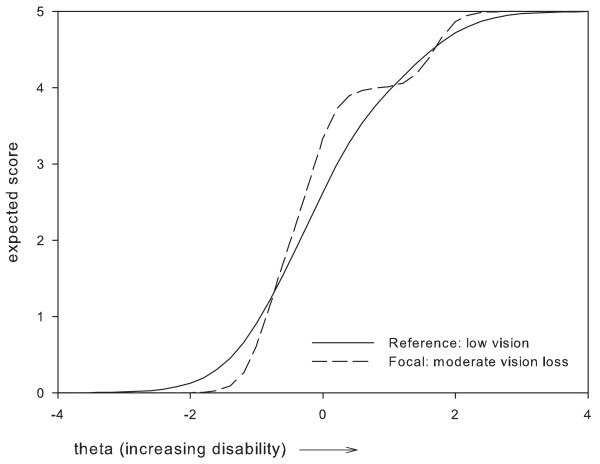
**Non-uniform DIF on item 3 'seeing steps or curbs' between vision category subgroups**.

**Figure 5:**
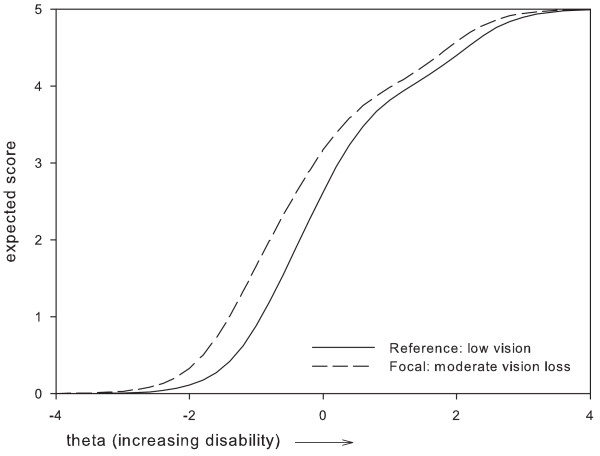
**Uniform DIF on item 7 'eyes getting tired' between vision category subgroups**.

**Figure 6:**
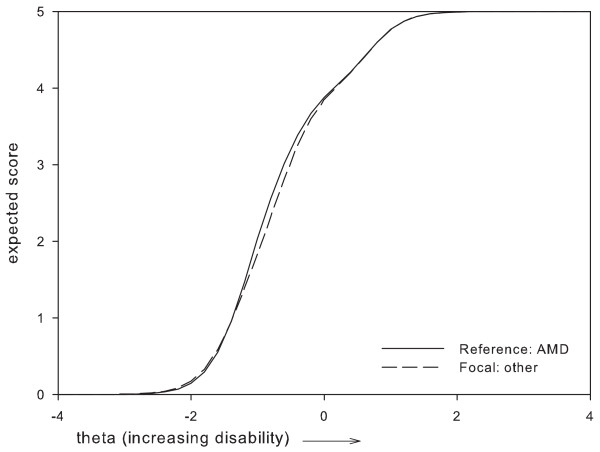
**Uniform DIF on item 19 'reading labels' between eye condition subgroups**.

Based on these results, two items which were perceived as most problematic were removed, i.e.: item 1 'Vision in general' and item 24 'Using tools'. Item 1 was removed because it presented with DIF between two subgroups, i.e. administration mode and gender, where the difference in expected item scores remained relatively large along a large part of the disability continuum (Figures 1, 2). For item 24, the maximum difference in expected scores exceeded 1 point between women and men, and, along a large part of the disability continuum (Figure 3). The difference in expected scores on items 3, 7 and 19 was not considered a problem, because differences between expected scores were not extreme, the place of this difference on the disability continuum was near the extremes (Figures 4, 5, 6), and DIF was only found for one subgroup variable per item.

Although most subgroups were comparable on most characteristics, differences were found between the LogMAR visual acuity subgroups, where patients with low vision/blindness significantly more often received assistance by someone to fill out the questionnaire (68%) than patients with mild vision loss (52%; p = 0.006). In addition, significantly less patients who went to the optometric service needed assistance with filling out the questionnaire (56%) compared to those who received multidisciplinary rehabilitation (70%; p = 0.012). Relatively more patients with age-related macular degeneration were in the 75+ age category (89%) than patients with other eye-conditions (59%; p < 0.001).

### Re-calibration after removing items

Table 3 presents the LVQOL items per dimension, calibrations and fit statistics without items 1 and 24. All items fit the GRM. The difficulty parameters (*β_1_-β_5_*) for the items which reflect the range of the underlying construct was between -2.17 and 2.55 for the "Basic aspects; -1.42 and 3.16 for "Mobility"; -1.06 and 3.48 for "Adjustment", and between -1.79 and 2.27 for "Reading and fine work". This means that the LVQOL items show reasonable variability with respect to endorsement of response categories by patients from the whole disability continuum.

**Table 3:** Item parameter estimates and fit statistics

	Item content per dimension	α	***β***_***1***_	*β_2_*	*β_3_*	*β_4_*	*β_5_*	**X**^**2**^	df	p
	**Basic aspects**									
6.	Seeing moving objects	2.06	-1.21	-0.66	0.13	0.66	2.55	19.6	20	0.49
7.	Eyes getting tired	2.61	-1.25	-0.96	-0.29	0.25	2.10	17.0	17	0.45
8.	Seeing the television	2.18	-1.69	-1.18	-0.34	0.21	1.98	17.1	18	0.52
9.	Glare (dazzled by lights)	1.81	-2.17	-1.80	-1.20	-0.51	1.76	24.8	19	0.17
10.	Getting right amount light	2.12	-1.83	-1.26	-0.37	0.22	2.32	13.0	20	0.88
	**Mobility**									
2.	Night vision inside house	1.66	-1.42	-0.88	0.32	0.85	3.16	24.6	22	0.31
3.	Seeing steps or curbs	3.56	-1.04	-0.68	-0.09	0.17	1.55	13.9	20	0.84
4.	Depth/distance perception	2.41	-0.80	-0.45	0.09	0.51	1.98	23.3	25	0.56
15.	Getting around outdoors	5.26	-1.07	-0.75	-0.14	0.22	1.72	7.3	18	0.99
16.	Crossing a road with traffic	3.35	-1.11	-0.69	-0.25	0.12	1.52	17.7	22	0.73
	**Adjustment**									
11.	Understand eye condition	1.26	-0.94	-0.47	0.22	0.68	3.48	17.0	21	0.71
12.	Unhappy situation in life	3.23	-0.63	-0.37	0.51	0.93	3.22	10.5	21	0.97
13.	Frustration with doing tasks	3.41	-1.06	-0.69	0.02	0.38	2.44	19.3	19	0.44
14.	Visiting friends and family	1.37	-0.44	-0.13	0.52	0.89	2.99	16.5	21	0.74
	**Reading and fine work**									
17.	Reading large print	2.14	-0.14	0.19	0.85	1.17	2.10	23.2	27	0.68
18.	Reading newspaper/books	3.98	-1.42	-1.06	-0.61	-0.26	0.92	23.7	25	0.54
19.	Reading labels	3.50	-1.79	-1.44	-0.94	-0.54	0.79	13.2	22	0.93
20.	Reading letters and mail	5.02	-1.28	-0.93	-0.36	0.02	0.98	21.9	22	0.47
21.	Finding out the time	1.91	-0.50	-0.16	0.59	1.13	2.27	25.8	29	0.64
22.	Writing	3.55	-1.04	-0.71	-0.17	0.17	1.06	21.2	27	0.78
23.	Reading own handwriting	3.17	-0.81	-0.41	0.11	0.51	1.31	24.5	26	0.55

Person distribution standard normal; *α *= item discrimination parameter; *β *= item threshold parameter;

X^2 ^= Chi-squared item fit statistic; df = degrees of freedom.

DIF analyses were repeated for "Adjustment" without item 1 on the subgroup variable gender. DIF for item 12 resolved at the p < 0.01 level. DIF analyses were repeated for "Reading and fine work" without item 24 on the subgroup variable eye condition. Uniform DIF remained for item 19 (G^2^(5) = 18.1; p < 0.01) between patients with age-related macular degeneration and patients with other eye conditions. However, the difference in expected scores remained small. Consequently, item 19 was not removed from this dimension.

**Figure 7:**
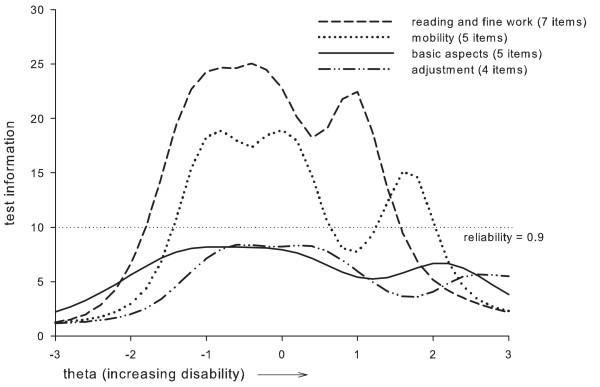
**Test information curves for the LVQOL dimensions**. The horizontal dash line is a reference line representing classical reliability = 0.9 [43,44]

Figure 7 presents the test information curves of the four dimensions of the LVQOL, providing information about precision of the dimensions across the disability continuum. The dimensions were less precise at the extremes; however, the whole disability spectrum was covered by the dimensions. At the highest point of the information curves, the lowest SEs are calculated. For "Reading and fine work", the highest information point was 25.0 (SE 0.20 for *θ_s _*= -0.4); for "Mobility" 18.9 (SE 0.23 for *θ_s _*= -0.8); for "Basic aspects" 8.2 (SE 0.35 for *θ_s _*= -0.8); and for "Adjustment" 8.4 (SE 0.35 for *θ_s _*= -0.6). Furthermore, the "Mobility" dimension showed a slight 'information dip' around a *θ_s _*of 1.0, but was still about equally informative as the "Basic aspects" and "Adjustment" dimensions.

Finally, the indices of subject separation were high for all dimensions: "Reading and fine work" (0.94); "Mobility" (0.91); "Basic aspects" (0.86); and "Adjustment" (0.83).

## Discussion

The purpose of this study was to assess some essential psychometric properties of the LVQOL using an IRT model. Special attention was paid to investigating DIF on relevant background variables. All items of the four LVQOL dimensions fit the GRM, also after two items were removed because of DIF. DIF was found on five items between subgroups of gender, visual acuity, administration modes and eye conditions. However, only item 1 'Vision in general' of the "Adjustment" dimension and item 24 'Using tools' of the "Reading and fine work" dimension were considered to be a problem. Item 1 had DIF between the administration mode subgroups and gender subgroups, where the difference in expected item scores remained relatively large along a large part of the disability continuum. Patients who self-administered the questionnaire responded lower to this item conditional on their disability level than patients who were assisted by a significant other, which was often a relative or spouse (91.3%; n = 183). Wolffsohn et al. found that patients who were assisted by someone reported higher disability levels measured with the LVQOL; they concluded that the subgroup which was assisted with administration had more vision loss and reduced contrast sensitivity than the self-report subgroup, but also suggested that the difference might reflect a negative bias introduced by the patient's relative [40]. An earlier study in which the psychometric quality of the Vision-related quality of life Core Measure was assessed in the same visually impaired patient group reported similar results with DIF present on two items [41]. Patients who were assisted had significantly more vision loss (mean logMAR Visual Acuity 0.74; SD 0.43) than patients who self-reported (mean 0.56; SD 0.90); this may explain why patients who were assisted scored higher on the item, conditional on their disability level. Similar to Wolffsohn et al., another plausible explanation was the nature of the relationship between the patient and the significant other who assisted with administration. The significant other may have (unconsciously) conveyed his/her personal opinion, or the patient's perception of the characteristics of the significant other may have prompted a socially-desirable response [42]. Furthermore, DIF on item 1 'Vision in general' between women and men was caused by a lack of responses in the highest response category.

There was a higher response to item 24 'Using tools' (e.g. using a hammer or threading a needle) of the "Reading and fine work" dimension by women than by men, conditional on the disability level. Because the difference in expected item scores was sufficiently large, and along a relatively large part of the disability continuum, it was decided to remove item 24.

A consequence of removing a differentially functioning item is that the psychometric quality of the underlying construct improves, i.e. vision-related quality of life and in particular the "Adjustment" and "Reading and fine work" dimensions. The four and seven remaining items on those dimensions, respectively, fit the GRM and DIF resolved for item 12 'Unhappy with situation in life'. Item 19 'Reading labels' continued to have DIF, but the difference in expected scores was small. The choice of removing an item with DIF is usually expressed by the difference in logits. A problem with polytomous item responses is that the difference in logits may vary for every threshold parameter, making the magnitude of DIF difficult to assess. Therefore, the difference in expected item scores was perceived as a helpful interpretation of the DIF magnitude [38]. Another consequence of improvement of the dimensions "Reading and fine work" and "Adjustment" might be that item invariance across occasions can be assumed. However, after removing item 24 'Using tools', the assumption of item parameter invariance across time points could still not be maintained for the "Reading and fine work" dimension (data not shown). Consequently, further investigation and confirmation in other longitudinal studies may be necessary. In contrast, after removing item 1 'Vision in general', item invariance was assured across occasions for the "Adjustment" dimension, indicating that the outcome on this dimension can be appropriately assessed. A limitation of the present study may be that the subsets on which DIF was investigated were rather small (N < 100 in two subsets). Differences in patient characteristics found between subsets may have been caused by limited numbers of patients.

Finally, the test information curves provided insight into the separate dimensions of vision-related quality-of-life. The "Reading and fine work" and "Mobility" dimensions were most informative for differentiating between patients' disability levels in terms of vision-related quality-of-life.

## Conclusion

The items of the LVQOL showed satisfactory item fit to the GRM; however, two items were removed because of DIF. The adapted (Dutch) LVQOL with 21 items is 'DIF-free' when relevant subgroups are considered, which means that the psychometric quality of the questionnaire has improved. Consequently, the LVQOL seems highly appropriate for use in heterogeneous populations of visually impaired patients.

## List of abbreviations

DIF: Differential item functioning; GRM: Graded response model; IRT: Item response theory; IRTLRDIF: Item response theory likelihood-ratio tests for differential item functioning; LVQOL: Low Vision Quality Of Life questionnaire; WLSMV: Weighted least squares with mean and variance correction.

## Competing interests

The authors declare that they have no competing interests.

## Authors' contributions

RMAVN drafted the manuscript and performed the statistical analyses; DLK gave advice and performed the statistical analyses, and helped to interpret the data; ML helped to draft and revise the manuscript; GHMBVR conceived of the study and its design; helped to draft the manuscript, and approved the final version to be published. All authors read and approved the final manuscript.

## Authors' information

RMAVN (PhD) is a psychologist and epidemiologist and has a special interest in the measurement of quality-of-life in the field of low vision. She received the Quality of Care Fellowship (200-2012) from the EMGO^+ ^Institute for Health and Care Research. DLK (PhD) is a statistician and is specialized in psychometrics and specifically in item response theory. ML (PhD) is a human movement scientist, epidemiologist and a former occupational therapist and researcher in the field of low vision. GHMBVR (PhD) is a professor of ophthalmology and holds a chair in the field of low vision.

## Pre-publication history

The pre-publication history for this paper can be accessed here:

http://www.biomedcentral.com/1471-2288/11/125/prepub
